# Thyroid Dysfunction as a Component of an Immuno-Metabolic Depression—A Possible Role of Gut Microbiota and a Rationale for Future Studies

**DOI:** 10.3390/cells15080723

**Published:** 2026-04-19

**Authors:** Karolina Michno, Mateusz Kapela, Dominik Strzelecki, Oliwia Gawlik-Kotelnicka

**Affiliations:** 1 Faculty of Medicine, Medical University of Lodz, 90-419 Lodz, Poland; karolina.michno@student.umed.lodz.pl (K.M.); mateusz.kapela@stud.umed.lodz.pl (M.K.); 2 Department of Affective and Psychotic Disorders, Medical University of Lodz, 92-216 Lodz, Poland; dominik.strzelecki@umed.lodz.pl

**Keywords:** depression, low-grade inflammation, metabolic syndrome, microbiota, thyroid diseases

## Abstract

**Highlights:**

**What are the main findings?**

- Increased intestinal permeability along with dysbiosis might contribute to dysregulation in both brain and thyroid activity.
- Depression and thyroid disorders might share common pathophysiological mechanisms and present with similar symptoms.

**What are the implications of the main findings?**

- Targeting gut health (e.g., restoring microbiota balance and improving intestinal barrier function) could potentially support both mental health and thyroid regulation.
- Clinicians should consider overlapping mechanisms when diagnosing and treating depression and thyroid disorders, as addressing one condition might influence the other.

**Abstract:**

Depression is one of the most prevalent psychiatric disorders worldwide, with a steadily increasing incidence and complex, multifactorial pathophysiology. Beyond classical neurochemical mechanisms, growing evidence points to the role of systemic low-grade inflammation and immuno-metabolic disturbances in its development. Gut microbiota dysbiosis has emerged as a key factor linking metabolic, immune, and neuroendocrine pathways, potentially exacerbating neuroinflammation and contributing to the onset and progression of depressive symptoms. Immune activation, which is a result of gut dysbiosis, may play a crucial role in the pathogenesis of immuno-metabolic depression. Thyroid dysfunction appears to be an important, yet insufficiently understood component of this network. Thyroid hormones play a crucial role in regulating metabolism, immune responses, and central nervous system function. Alterations in thyroid function, even within subclinical ranges, have been associated with mood disturbances and may share common inflammatory and metabolic pathways with depression. Furthermore, emerging data suggest that gut microbiota may influence thyroid hormone metabolism, including deiodinase activity, linking dysbiosis with thyroid axis dysregulation. Despite these insights, the integrated interactions between thyroid function, gut microbiota, metabolic syndrome, and inflammation in depression remain largely unexplored. This review explores current evidence to highlight gaps in existing research and synthesizes current knowledge, aiming to clarify mechanisms underlying immuno-metabolic depression. Understanding these relationships may provide a rationale for redefining depression as an immuno-metabolic disorder and support the development of more integrative therapeutic strategies targeting not only the brain, but also the gut-thyroid axis.

## 1. Introduction

Depression is a common psychiatric disorder, affecting approximately 280 million people worldwide, according to the WHO [1]. Its prevalence has been rising, with increasing episodes of mild-to-severe and moderate-to-severe depression noted over time [2]. This condition is a leading cause of global disease burden, marked by persistent mood disturbances, functional impairment, and heightened risks of suicide and cardiovascular morbidity [3,4]. Given its significant impact, understanding the underlying mechanisms of depression is crucial for the development of more effective and targeted therapeutic strategies.

Although traditionally explained by monoaminergic and neuroanatomical models, depression is increasingly understood as a systemic disorder involving immune, metabolic, and neuroendocrine dysregulation [5,6].

Emerging research has begun to uncover the pivotal role of the immune and metabolic systems in the pathophysiology of depression, leading to the concept of immuno-metabolic depression [7]. This perspective emphasizes the interplay between chronic inflammation, altered metabolic processes, and dysregulation of neuroendocrine pathways in the development of depressive symptoms.

Psychiatric disorders frequently co-exist with metabolic syndrome and cardiovascular diseases, suggesting that shared pathophysiological mechanisms underpin these comorbidities [8,9]. Metabolic disturbances such as obesity, insulin resistance, and dyslipidemia are often accompanied by alterations in thyroid function, including both overt and subclinical hypothyroidism, which can further exacerbate metabolic dysregulation and influence mood [10,11]. This overlap highlights the clinical relevance of immuno-metabolic depression, as it provides a framework for understanding how metabolic, endocrine, and immune dysregulation may contribute to psychiatric symptomatology. Previous studies have shown that depression is tightly correlated with obesity, and this connection is bidirectional. People with depression have an increased chance of being obese (58%), and obese people have an increased risk of developing depression (55%) [12]. Additionally, individuals with depression and anxiety have an increased risk of developing thyroid diseases, with more severe depression and anxiety correlating with a higher associated risk [13].

Within this framework, pro-inflammatory cytokines have been shown not only to affect mood regulation but also to disrupt key metabolic pathways, including insulin sensitivity, glucose metabolism, and lipid homeostasis. Metabolic disturbances—such as obesity, insulin resistance, and alterations in gut microbiota—may both contribute to depressive disorders and be exacerbated by depression itself, reflecting a bidirectional relationship [14]. In this context, the microbiota–gut–brain axis has emerged as a central mechanism, linking gut microbial alterations with central nervous system function and integrating immune, metabolic, and neuroendocrine signaling pathways involved in mood regulation. Considering these complex interactions, the clinical overlap of metabolic and psychiatric disorders becomes even more relevant. Despite growing evidence linking depression, metabolic syndrome, and thyroid dysfunction, the precise mechanisms connecting these conditions remain incompletely understood. Importantly, the current literature is dominated by observational studies, which limit causal inference and do not allow for clear differentiation between cause and consequence. Moreover, the potential role of gut microbiota as a mediator of immune-metabolic dysregulation remains insufficiently characterized, particularly in the context of integrative, multi-system models of depression. Recognizing these gaps, the present study aims to critically review current findings on these interrelationships, identify gaps in the literature, and highlight potential pathways through which dysbiosis, low-grade inflammation, thyroid dysfunction, and metabolic disturbances may intersect and contribute to depression. By synthesizing existing evidence, we aim not only to summarize current knowledge but also to critically evaluate the quality of available studies, identify inconsistencies, and highlight key gaps that should be addressed in future mechanistic and longitudinal research.

## 2. Materials and Methods

### 2.1. Literature Search Strategy

A comprehensive literature search was conducted across the PubMed, Scopus, and Web of Science databases. The review covered studies published between 2000 and 2026 to capture both foundational research and recent developments in the field. A combination of keywords and their synonyms was used, including terms related to depression, obesity, metabolic syndrome, inflammation, thyroid disorders, and gut microbiota.

### 2.2. Eligibility Criteria

Studies were considered eligible if they were published in peer-reviewed journals, available in English, and examined the relationship between depression and metabolic, endocrine, or gut microbiota-related factors. Both studies involving human participants and relevant animal models were included. Publications such as conference abstracts, editorials, and case reports were excluded, as well as studies without full-text availability or those not directly relevant to the scope of this review.

### 2.3. Study Selection and Data Extraction

Titles and abstracts were screened to identify potentially relevant studies. Full texts of selected articles were then assessed for eligibility based on predefined inclusion and exclusion criteria. Relevant data were extracted from the included studies, focusing on study design, population characteristics, and key findings on interactions among depression, metabolic syndrome, thyroid function, inflammation, and gut microbiota. The findings were synthesized narratively due to heterogeneity in study designs and outcome measures.

## 3. Immuno-Metabolic Depression

Immuno-metabolic depression (IMD) can be conceptualized as a biologically grounded dimension of depression, in which immune and metabolic dysregulations have been implicated in its pathophysiology. Rather than presenting a clearly separated subtype, IMD can be viewed as a specific profile within the broader spectrum of depressive disorders, reflecting their clinical and biological diversity. This conceptualization provides a helpful basis for connecting clinical observations to underlying mechanisms discussed previously [15].

This profile is most consistently associated with symptoms related to altered energy balance, including weight gain, increased appetite, fatigue, and reduced energy. These features are proposed to result from interactions among low-grade inflammation, metabolic disturbances, and altered neuroendocrine pathways regulating energy balance [15].

Recognizing the distinct biological background of this form of depression may facilitate the development of more targeted and effective therapeutic strategies. Such an approach could improve treatment outcomes by moving beyond uniform treatment models and toward more personalized interventions.

In this context, the overlap between IMD and metabolic syndrome appears particularly relevant, as both share common inflammatory and metabolic pathways. This supports the conceptualization of metabolic syndrome as a relevant component of immuno-metabolic depression, which will be discussed in the following section.

Importantly, this phenotype may also have therapeutic implications, as growing evidence indicates that patients with an immuno-metabolic profile of depression—particularly those with elevated inflammatory markers—in meta-analyses tend to show reduced responsiveness to standard antidepressant therapies [16,17]. Additionally, recent evidence suggests that metabolic dysregulation may also influence the effectiveness of adjunctive treatments, as patients with impaired metabolic status showed a diminished response to probiotic interventions in depression [18]. This brings out the need for more targeted, multimodal treatment strategies that address underlying immune and metabolic dysfunction.

### 3.1. Depression and Metabolic Syndrome

Metabolic syndrome (MetS), defined by the coexistence of obesity, hypertension, hyperglycemia, and dyslipidemia, represents a major and growing public health concern in Europe [19,20]. Its prevalence has been estimated at approximately 24.3%, with a marked increase with age, from 3.7% in individuals aged 20–29 years to over 30% in those aged 70 years or older [21].

According to Eurostat study prevalence of overweight in Europe (body mass index (BMI) ≥ 25 kg/m^2^) in people aged 16 years or over (as of the year 2022) was between 31.3% (Italy) and 56.7% (Latvia) for females and between 51.5% (France) and 69.4% (Croatia, Malta and Slovakia) for males. Epidemiological data further indicate a high burden of excess body weight across Europe. According to Eurostat (2022), the prevalence of overweight (BMI ≥ 25 kg/m^2^) ranges from approximately 31% to 57% in women and 51% to nearly 70% in men, depending on the country. Obesity (BMI ≥ 30 kg/m^2^) also remains highly prevalent, with the highest rates reported in Romania and Poland among men (38% and 32%, respectively), and in Romania, Ireland, and Croatia among women (up to 32%) [22]. These data highlight the widespread nature of metabolic disturbances in European populations and underscore their relevance in the context of immuno-metabolic disorders.

Unhealthy dietary patterns and reduced physical activity contribute to visceral adiposity, which drives the overproduction of inflammatory molecules. Furthermore, insulin resistance increases lipolysis and promotes overproduction of very low-density lipoprotein (VLDL), thereby increasing free fatty acid levels in the bloodstream. Leptin levels increase in obesity, stimulating immune cells by activating the type 1 T helper cell (Th1) pathway [23], which can influence the immune system and increase inflammation. Chronic low-grade inflammation seen in obesity may contribute to thyroid dysfunction, such as autoimmune thyroid disorders like Hashimoto’s thyroiditis [24].

Obesity has also been shown to influence thyroid hormone levels. It is often associated with changes in the hypothalamic-pituitary-thyroid (HPT) axis, leading to slightly elevated TSH levels even in individuals without overt thyroid disease [24].

Several studies have demonstrated that obesity may be associated with significant alterations in gut microbiota composition. One key finding is a reduction in *Akkermansia muciniphila*, a mucin-degrading bacterium that plays an important role in maintaining gut barrier integrity and modulating host metabolism [25]. Decreased abundance of *A. muciniphila* has been correlated with low-grade systemic inflammation and metabolic dysfunction [26]. Furthermore, obese individuals often exhibit an increased *Firmicutes*-to-*Bacteroidetes* (F/B) ratio [27,28]. A decrease in Bacteroidetes may impair the normal metabolic and anti-inflammatory functions of the gut microbiome [29]. Together, these microbiota changes potentially create a microenvironment that promotes weight gain, insulin resistance, and systemic inflammation. However, some studies emphasize the high interindividual variability and the influence of numerous factors on study outcomes, underscoring the need for further research in this field [30].

Importantly, despite numerous studies linking gut microbiota composition with obesity, the causal relationship remains uncertain. Many findings are based on cross-sectional analyses, which do not allow for temporal or mechanistic conclusions. Moreover, significant interindividual variability, dietary differences, and methodological heterogeneity across studies limit reproducibility. Therefore, future research should focus on standardized, longitudinal designs and functional analyses of microbial metabolites rather than solely taxonomic composition.

All the above-mentioned conditions have been proven to coexist with depression, generally more often than usual. Prevalence of depression amongst centrally obese individuals (defined as BMI > 28 kg/m^2^ and waist circumference (WC) > 90 cm in men and >85 cm in women) is confirmed to be higher than among the rest of the population (17.83% in centrally obese patients and 12.6% in non-obese participants) [31]. Moreover, the relative risk of metabolic syndrome is 1.57 times higher amongst psychiatric patients with MDD than in the healthy control group [32].

Importantly, there is a bidirectional relationship between depression and metabolic syndrome. A less active lifestyle, unhealthy dietary habits, and poorer accessibility of medical care for depressed patients may lead to obesity. The hyperactivity of the HPA axis is also an important contributing factor that, through adipogenesis, leads to increased lipid storage and therefore visceral fat accumulation. Additionally, some antidepressants are known to present side effects such as weight gain, which is associated with the use of certain tricyclic antidepressants, monoamine oxidase inhibitors, and selective serotonin reuptake inhibitors [33]. Furthermore, tricyclic antidepressants, along with noradrenergic and serotonergic (NS) working antidepressants, have been linked with increased risk of hypertension [34], which is yet another component of metabolic syndrome.

On the other hand, adipose tissue contributes to chronic low-grade inflammation by increasing production of pro-inflammatory cytokines and by promoting oxidative stress, which may further exacerbate systemic metabolic dysfunction [23,35].

Consistent evidence from large systematic reviews and meta-analyses indicates a modest but clear bidirectional association between depression and MetS, with moderate strength of evidence due to predominance of observational studies and limited causal interference [36,37]. Importantly, recent randomized trials suggest that aerobic exercise interventions can improve psychosocial functioning and metabolic parameters in individuals with MetS, indicating that behavioral interventions may modulate shared metabolic and inflammatory pathways [38]. Interestingly, running therapy led to greater reductions in pro-inflammatory cytokines and metabolic dysregulation compared with antidepressants, emphasizing its capacity to target the biological mechanisms underlying immuno-metabolic depression [39].

Taken together, current evidence supports a bidirectional relationship between depression and metabolic syndrome; however, the underlying mechanisms remain incompletely understood. The predominance of observational studies limits causal inference and makes it difficult to distinguish behavioral from biological contributors. Moreover, despite well-established associations, there is a lack of integrative studies simultaneously addressing metabolic, inflammatory, and neuroendocrine pathways within a unified mechanistic framework. This represents a significant gap in the current understanding of immuno-metabolic depression and highlights the need for more comprehensive, longitudinal, and mechanistic research.

### 3.2. Depression and Inflammation

Although cytokines are produced peripherally, they can influence brain function. Cytokines can cross the blood–brain barrier directly via the saturable transport systems or indirectly via microglia activation. Both of these ways are consequent in altering brain function—reduced synthesis and reuptake of some neurotransmitters, as well as oxidative stress leading to neurotoxicity [40].

There are various ways that low-grade inflammation affects the brain signaling, which may lead to depression. The kynurenine pathway of tryptophan metabolism is one of the affected subjects. In the presence of excessive levels of pro-inflammatory cytokines, tryptophan is converted to the neurotoxic quinolinic acid, which also results in a shortage of serotonin. Furthermore, low-grade systemic inflammation can also disrupt dopaminergic transmission by impairing the cofactor tetrahydrobiopterin (BH4), which is crucial for the key enzyme in the conversion of tyrosine to L-DOPA [40]. As evidence seems to indicate, motivational anhedonia and alterations in reward-seeking behavior (so-called “sickness behavior”-similar to symptoms occurring in depression) might be directly caused by abnormalities of mesolimbic dopamine signaling [41].

**Figure 1:**
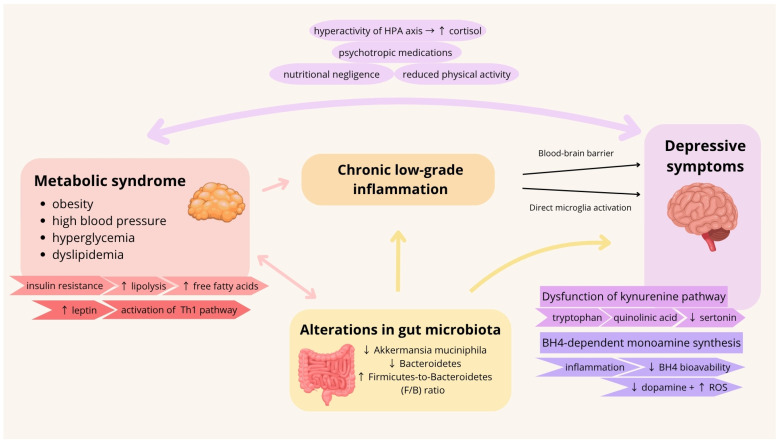
Illustration of mechanisms linking metabolic syndrome, alterations of gut microbiota, and depression. ↑—elevated; ↓—decreased; BH4—tetrahydrobiopterin; HPA—hypothalamic-pituitary-adrenal axis; ROS—reactive oxygen species.

Although a growing body of evidence supports the role of inflammation in depression, it should be noted that many studies rely on peripheral inflammatory markers, which may not fully reflect central nervous system processes. Furthermore, heterogeneity in patient populations and diagnostic criteria complicates interpretation. This highlights the need for studies integrating peripheral biomarkers with neuroimaging and functional assessments. Figure 1 summarizes the mechanisms linking metabolic syndrome, gut microbiota, and depression, mediated by chronic low-grade inflammation.

## 4. Thyroid Dysfunction

### 4.1. Thyroid Dysfunction and Metabolic Syndrome

The prevalence of undiagnosed thyroid dysfunction in Europe was assessed in 7 studies, with a mean result of 6.71% of the population, and over 80% of those dysfunctions were subclinical [42]. The most common thyroid dysfunctions are hypothyroidism and hyperthyroidism, and the main reason for these conditions in iodine-sufficient countries is Hashimoto’s thyroiditis [43]. Both of these conditions may be subclinical, meaning that when thyrotropin (TSH) serum levels are abnormal, the serum concentrations of free thyroxine (fT4) and triiodothyronine (fT3) are within a normal range.

Several mechanisms may link thyroid dysfunction with metabolic syndrome. Thyroid hormones influence glucose and lipid metabolism by increasing gluconeogenesis and its output by the liver and its uptake and utilization in muscles, as well as higher lipolysis in white adipose tissue. Furthermore, thyroid hormones control food intake by increasing leptin release. Subsequently, hypothyroidism might lead to insulin resistance due to insufficient glucose transport and utilization in peripheral tissues [44].

It remains unclear whether elevated TSH and T3 levels observed in obese individuals are a consequence of weight gain or rather the cause of it. As leptin is a neuroendocrine regulator of the hypothalamic–pituitary–thyroid (HPT) axis, it can directly regulate TRH gene expression in the paraventricular nucleus and in the arcuate nucleus. In obese patients, TSH levels positively correlate with leptin levels, both of which are elevated. Nevertheless, no correlation has been found between energy expenditure and increased levels of fT3 and TSH in euthyroid obese individuals, not proving the theory of hyperleptinemia being a mechanism aiming to reduce obesity. Therefore, evidence indicates that a rise in hyperthyrotrophinemia might be secondary to weight gain rather than the cause of it (reviewed in Biondi, 2024 [45]). Overall, while associations between thyroid dysfunction and metabolic disturbances are well documented, the directionality and causality of these relationships remain unclear. Many conclusions are based on observational data, and interventional studies are scarce. Future research should focus on mechanistic pathways linking thyroid hormones with metabolic and neuropsychiatric outcomes.

### 4.2. Correlation of Depression and Thyroid Diseases

An association between depressive symptoms and thyroid disorders has been demonstrated in multiple observational studies. Thyroid function tests (especially TSH) are laboratory examinations performed amongst patients diagnosed with MDD, as hypothyroidism may resemble depression. Patients with overt hypothyroidism present symptoms regarding mood, such as apathy and slowing of thought and speech, a decrease in concentration, memory function, and perception, followed by fatigue [46]. Furthermore, a deficiency of thyroid hormones may reduce prefrontal cortex activity and decrease gray matter volume in the right middle frontal gyrus, thereby contributing to impaired mood regulation [47]. In this regard, there is an elevated risk that patients with hypothyroidism might be diagnosed with depression instead of thyroid disorder [48].

However, it is inconclusive whether variation in normal-range thyroid-related hormones (subclinical) presents a risk factor for MDD. Large Mendelian randomization analyses on 500,199 individuals found no correlation between variations in normal-range TSH and fT4 levels and risk of MDD, nor minor depressive symptoms [49]. On the other hand, a large retrospective cohort study demonstrated an increased risk of developing depressive symptoms among euthyroid female subjects in the highest tertile of TSH levels, in comparison with subjects with the lowest tertile [50]. These findings highlight the importance of sex-specific analyses in future research. Moreover, a large population-based study suggests that suppressed TSH levels, reflecting subclinical hyperthyroidism, are associated with modestly increased risk of subclinical depression [51]. Interestingly, a retrospective observational study on adolescents found that those with bipolar and unipolar depression had significantly higher mean serum TSH levels than those diagnosed with conduct disorders and mixed disorders of conduct and emotions [52], suggesting a potential connection between thyroid function and depressive disorders in particular.

Zhou et al.’s retrospective study found lower TSH, fT3, and fT4 levels in MDD patients than in healthy controls, with no difference in the prevalence of overt or subclinical thyroid dysfunction between the two groups [53]. In this regard, it remains unclear whether depressive symptoms should be associated with minor alterations in thyroid function. This study is limited by its cross-sectional design and potential misclassification of the control group, as the absence of thyroid and psychiatric disorders was not fully verified. Additionally, lack of detailed data on antidepressant use in MDD patients and incomplete thyroid function assessment (e.g., absence of T3, T4, and autoantibody measurements) may have introduced confounding and limited interpretation of the findings.

It has been suggested that weight loss following bariatric surgery is associated with decreases in TSH levels. Janssen et al. demonstrated that Roux-en-Y gastric bypass induced significant weight loss in 61 patients with subclinical hypothyroidism, resulting in a decrease in TSH and normalization of thyroid function in approximately 87% of cases at 12 months postoperatively [54]. These findings suggest that weight reduction may normalize HPT axis activity, which could contribute to improvements in depressive symptoms associated with subclinical hypothyroidism. Despite these promising observations, current evidence is largely limited to observational studies. Investigations simultaneously assessing weight loss, thyroid hormone normalization, and changes in depressive symptoms are needed to clarify the complex interactions between metabolic dysregulation, thyroid function, and depression.

Although serum TSH and peripheral thyroid hormone levels may appear normal in patients with depression, a phenomenon known as brain hypothyroidism could be present. This condition is characterized by reduced intracerebral fT3 levels due to impaired conversion of fT4 to fT3 by type II deiodinase in the brain, potentially leading to local thyroid hormone deficiency that affects mood and cognitive function [55]. Supporting this concept, neuroimaging evidence indicates that patients with comorbid MDD and subclinical hypothyroidism exhibit altered activity in brain regions involved in mood regulation, including the frontal cortex, insula, and thalamus. Notably, serum TSH levels were associated with changes in insular activity, which correlated with the severity of depressive symptoms [47].

Taken together, current evidence does not allow for a definitive conclusion regarding the role of subtle thyroid hormone variations in the pathogenesis of depression, highlighting the need for well-designed longitudinal and mechanistic studies to clarify whether subtle thyroid hormone alterations play a causal role in depression or represent an epiphenomenon.

Interestingly, elevated CSF concentrations of TRH (thyrotropin-releasing hormone) were observed in depressed patients. It has been hypothesized that increased TRH release may compensate for reduced serotonergic activity, leading to downregulation of pituitary TRH receptors and a blunted TSH response [11]. Beyond this compensatory mechanism, TRH may also act as a central neuromodulator influencing monoaminergic systems, particularly dopaminergic and serotonergic pathways, thereby linking HPT axis dysregulation with broader neurochemical alterations observed in depression [56]. Given that TRH may exert antidepressant effects [57,58], further investigation of the hypothalamic-pituitary-thyroid axis could provide valuable insights into the neuroendocrine mechanisms underlying depression.

A recent meta-analysis by Loh et al. evaluated the impact of levothyroxine therapy on depressive symptoms in patients with coexistent subclinical hypothyroidism. The results indicate that levothyroxine does not consistently alleviate depressive symptoms, potentially due to short intervention periods, the type of therapy, or brain hypothyroidism [55]. In a limited case series, augmentation with liothyronine improved depressive symptoms in a small group of patients who did not respond to levothyroxine as monotherapy [59]. This suggests that directly targeting intracerebral fT3 deficiency may be necessary for effective treatment in cases of brain hypothyroidism. However, subsequent studies reported inconsistent results, and the evidence for an added effect of combined levothyroxine/liothyronine therapy on mood and cognitive performance compared with levothyroxine monotherapy remains inconclusive [60]. Most of these studies were conducted in relatively small patient groups, limiting the applicability of the findings and highlighting the need for confirmation in larger clinical trials.

**Figure 2:**
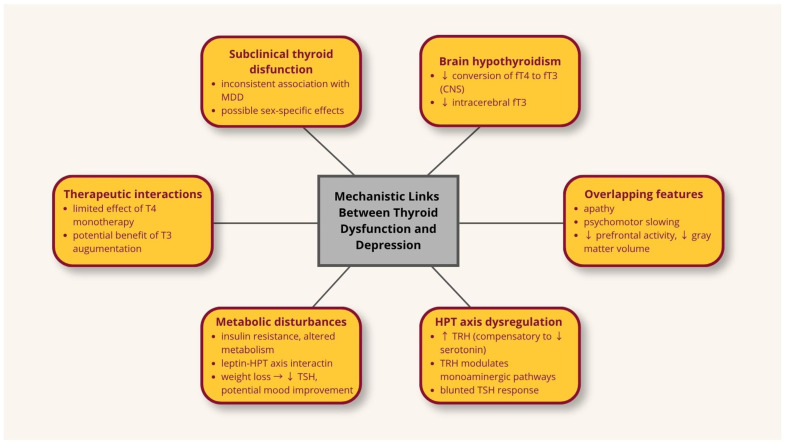
Summary of the current knowledge on the relationship between thyroid dysfunction and depression. ↑—elevated; ↓—decreased; CNS—central nervous system; HPT—hypothalamic-pituitary-thyroid; MDD—major depressive disorder; T3—liothyronine; fT3—free triiodothyronine; T4—levothyroxine; fT4—free thyroxine; TRH—thyrotropin-releasing hormone; TSH—thyroid-stimulating hormone.

Although associations between thyroid dysfunction, metabolic disturbances, and depression are well documented, their directionality and underlying mechanisms remain unclear. Current evidence is limited by a lack of integrative studies that simultaneously evaluate thyroid function, metabolic status, and depressive symptoms, thereby restricting a comprehensive understanding of their interplay within the immuno-metabolic framework. Future research should therefore prioritize longitudinal and interventional designs to clarify how subtle alterations in thyroid hormone levels interact with systemic inflammation, neuroendocrine signaling, and behavioral outcomes in immuno-metabolic depression. Current concepts regarding the mechanisms linking thyroid dysfunction and depression are presented in Figure 2.

## 5. Gut Microbiota and Dysbiosis

### 5.1. Low-Grade Inflammation as a Result of Dysbiosis

Dysbiosis is defined as an imbalance in bacterial composition, alterations in bacterial metabolic activity, or improper distribution of microbiota within the gut. The crucial function of the healthy gut microbiota is to maintain and regulate an intestinal barrier, and this bacterial composition may be affected by diet, medication use, age, or smoking cigarettes [61,62].

Increasing evidence suggests that dysbiosis leads to a condition known as leaky-gut syndrome, characterized by increased intestinal barrier permeability. As a result, underlying immune components are activated, leading to local inflammation [63]. Consequently, bacteria might become translocated through the impaired barrier to the bloodstream. On the surface of Gram-negative bacteria, there are usually lipopolysaccharides (LPS), which bind to LPS-binding protein in circulation. Such a complex triggers the immune system, which produces an increasing amount of cytokines [40]. Interestingly, levels of antibodies against the LPS in depressed patients are notably higher than those of healthy patients [64].

It has also been found that iodine uptake in thyroid cells is primarily mediated by the sodium/iodide symporter (NIS). It is hypothesized that modulation of thyroidal iodine metabolism via alterations in NIS expression and functional activity may represent a potential mechanistic pathway through which LPS and short-chain fatty acids (SCFAs), derived from the gut microbiota, exert their effects [65]. LPS may also directly influence thyroid cells by enhancing the expression of thyroglobulin and NIS genes in response to TSH, as demonstrated in a rat thyroid cell line [66].

Intestinal barrier dysfunction can be assessed through biomarkers such as lipopolysaccharide-binding protein (LBP) in blood, intestinal fatty acid-binding protein (I-FABP), and zonulin in stool [67].

Gut microbiota synthesize short-chain fatty acids (SCFAs) during the fermentation of cellulose. The acetic acid, propionic acid, and butyric acid are the most abundant of SCFAs [68]. Besides impacting the blood–brain barrier, they exert multiple systemic effects, including modulation of immune responses and maintenance of intestinal barrier integrity, thereby influencing host metabolic and inflammatory homeostasis [69,70].

The intestine contains a large population of CD4+ regulatory T cells (Treg) that maintains homeostasis at the mucosal surfaces, where intestinal bacteria and metabolites play a significant role in the generation or maintenance of this Treg population [71]. SCFAs are among the major bacterial products linked to the expansion and suppressive function of Treg cells. It has been shown that SCFAs can modulate Treg expansion and function by altering the function of antigen-presenting cells (APCs). However, SCFAs have also been shown to induce pro-inflammatory Th1 and Th17 cells during an active immune response [72].

There are several ways in which the gut microbiome might influence metabolic syndrome risk. Firstly, healthily composed intestinal bacteria might be protective against obesity, as suggested by a study on rats. In rodents fed oligofructose, weight gain, food intake, and serum triglyceride accumulation were smaller after subsequently implementing a high-fat diet than in the control group [73]. Additionally, in other studies, mice transplanted with fecal microbiota from obese twins experienced greater weight gain than those transplanted with microbiota from non-obese twins [74].

Moreover, dysbiosis might disrupt the circadian rhythm [75], which, in turn, promotes weight gain by elevating ghrelin and reducing leptin levels, thereby increasing energy intake [76]. The evidence above indicates the influence of gut microbiota on obesity, an essential element of metabolic syndrome.

Despite promising mechanistic insights, much of the current evidence is derived from animal models, which limits direct translation to human physiology. Additionally, variability in microbiome assessment methods and a lack of standardized biomarkers pose significant challenges for clinical applications. Future studies should prioritize human interventional designs and functional microbiome analyses.

### 5.2. Correlation of Microbiota and Thyroid Function

There is growing appreciation that, by maintaining immune homeostasis and metabolic health, the microbiota plays a significant role in thyroid health [77]. In patients with autoimmune thyroid diseases (AITDs), dysbiosis is frequently observed [78]. Dysbiosis might result in reduced SCFAs levels [79]. These alterations may contribute to immune dysfunction, as an imbalanced microbiota can promote pro-inflammatory responses and disrupt regulatory T-cell activity, thereby exacerbating autoimmunity [71]. Growing evidence suggests a correlation between thyroid dysfunction and gut microbiota [80]. There are several areas of overlap between these two topics, as described below.

Firstly, systematic reviews and meta-analyses have reported reduced microbial diversity and lower abundances of beneficial bacteria, such as *Bifidobacterium* and *Lactobacillus*, in AITD patients compared to healthy controls [78]. Studies suggest that these changes may contribute to the synthesis of antibodies that cross-react with thyroperoxidase and thyroglobulin through molecular mimicry, thereby promoting the development of autoimmune thyroid diseases [81].

Bile acids, which are bacterial metabolites generated from bile salts, have been suggested to influence energy metabolism through modulation of TSH-related pathways [82], indicating a potential gut-thyroid interaction. In a cross-sectional study by Song et al., lower total serum bile acid levels were observed in patients with subclinical hypothyroidism and were inversely associated with TSH concentrations. Furthermore, experimental data have shown that microbiota-derived metabolites such as n-butyrate can modulate thyroid hormone signaling by altering nuclear T3 receptor availability and enhancing T-3 dependent gene expression via epigenetic mechanisms [72]. These findings highlight a potential gut-thyroid axis in metabolic regulation. In Graves’ disease, an increase in *Bacteroidetes* and a decrease in *Firmicutes* have been noted [83]. At the same time, Hashimoto’s thyroiditis is often associated with increased gut permeability, allowing microbial antigens to interact with the immune system and trigger chronic inflammation. However, interpretation of these findings is limited by the fact that a substantial proportion of patients received antithyroid treatment, which may influence microbiota composition. Despite this, the study remains highly valuable for its large, multicenter European cohort, which enhances the robustness and generalizability of the observed associations.

Altered levels of SCFAs associated with dysbiosis may underlie autoinflammatory diseases, such as Hashimoto’s thyroiditis (HT) [84]. Recent studies have shown that individuals with HT have significantly lower butyrate levels compared to healthy controls. This reduction correlates with elevated pro-inflammatory cytokines, such as IL-6 and TNF-α, which further exacerbate thyroid tissue inflammation and dysfunction [77].

The role of SCFAs in thyroid dysfunction is poorly understood, and further studies are required to elucidate the links between the gut microbiota and thyroid function. Such mechanisms underline the potential role of the gut in modulating thyroid autoimmunity.

Taken together, current findings suggest a potential interaction between gut microbiota and thyroid function; however, evidence remains fragmented and largely associative. Further studies are required to determine whether alterations in the microbiota are a cause or consequence of thyroid dysfunction.

### 5.3. Role of Microbiota in the Pathomechanism of Immuno-Metabolic Depression

As a final component of the gut–thyroid–brain axis, it is essential to emphasize the direct role of the gut microbiota in regulating mood and depressive symptomatology. While previous sections outlined immune and metabolic consequences of dysbiosis, emerging evidence indicates that gut microbiota also influences central nervous system function through distinct neurobiological pathways.

Gut microbiota affects the central nervous system through multiple interconnected pathways, forming the microbiota–gut–brain axis.

Dysbiosis, defined as an imbalance in the gut microbial composition and function, can trigger a cascade of effects that contribute to depressive symptoms. Beyond peripheral immune activation described earlier, gut microbiota can modulate brain function through neuroendocrine, metabolic, and neural signaling pathways, leading to altered neurotransmitter metabolism, impaired neurogenesis, and oxidative stress—all of which are implicated in the pathophysiology of depression [40,70].

In addition to immune-mediated effects, gut dysbiosis can influence neuroendocrine and neural signaling pathways. For instance, microbial metabolites and neurotransmitter precursors produced in the gut modulate the hypothalamic–pituitary–adrenal (HPA) axis, affecting stress response and cortisol regulation [75,76]. Dysbiosis can also alter the synthesis of tryptophan and other amino acids that serve as precursors for serotonin and dopamine, thereby directly impacting mood and reward-related brain circuits [85]. Moreover, changes in microbial diversity and metabolic activity can influence vagal nerve signaling, providing another route by which gut composition affects brain function and emotional regulation [86].

Recent research has expanded our understanding of how gut microbiota composition affects depressive disorders, with mechanisms extending beyond general immune activation. Several large-scale human studies and systematic reviews have identified consistent differences in gut microbial profiles between individuals with depression and healthy controls, although findings on overall diversity remain mixed.

For example, a 2025 systematic review reported that patients with depressive disorders tend to have enriched pro-inflammatory bacterial taxa (e.g., *Actinobacteria*, *Proteobacteria*) and reduced anti-inflammatory SCFA-producing genera, despite inconsistent alpha and beta diversity results, partly due to diet and medication confounders [87].

SCFAs are among the microbial metabolites most consistently implicated in depression and are also strongly associated with visceral obesity and other metabolic disturbances. These compounds can cross the blood–brain barrier, modulate neurotransmitter systems such as serotonin, dopamine, and GABA, and exert anti-inflammatory and neuroprotective effects by reducing microglial activation and oxidative stress [88].

Animal studies have shown that supplementation with SCFA-producing prebiotics or direct SCFA administration alleviates depressive-like behaviors, whereas dysbiosis resulting in reduced SCFA production correlates with increased vulnerability to stress and depressive symptoms [89]. Human observational studies also indicate that lower fecal butyrate levels are associated with higher severity of depressive symptoms [70].

Importantly, gut dysbiosis may be associated with both decreased production of SCFA-producing bacteria and, in some contexts, increased fecal SCFA concentrations due to impaired absorption. Additionally, SCFAs-related alterations should not be interpreted solely as changes in absolute concentrations, as they also involve shifts in the relative proportions of individual SCFAs (e.g., acetate, propionate, and butyrate) within the total SCFAs pool, as well as changes in their mutual balance, reflecting complex microbial metabolic activity [18,90].

Interventional studies further support the role of gut microbiota in depression. Meta-analytic evidence indicates that administration of probiotics, prebiotics, and synbiotics can result in modest but statistically significant improvements in depressive symptoms, suggesting that modulation of microbiota composition may have therapeutic potential [91]. Emerging clinical data further indicate that probiotic supplementation may also reduce systemic inflammatory markers, such as C-reactive protein (CRP), particularly in individuals with elevated baseline inflammation or those receiving antidepressant treatment, supporting their immunomodulatory role [92]. However, effect sizes in interventional studies remain modest and heterogeneous, limiting immediate clinical applicability.

Experimental fecal microbiota transplantation (FMT) studies, though still emerging, have shown that transferring healthy gut microbial communities into depressed subjects may enhance response to conventional antidepressant treatment and reduce depressive scores more than medication alone, pointing to a causal role of specific bacterial profiles in mood regulation [93].

Overall, although accumulating evidence supports the involvement of gut microbiota in depression, the field is still limited by substantial heterogeneity across studies, including differences in populations, analytical techniques, and confounding factors such as diet and medication use. Importantly, most human studies remain observational, making it difficult to establish causality. Future research should focus on integrative, multi-omics approaches and well-controlled interventional studies to better define the therapeutic potential of microbiota modulation in depression.

To integrate the above-described mechanisms, Table 1 summarizes key microbiota-related alterations and their associated metabolic, inflammatory, and neuroendocrine consequences across selected clinical conditions.

## 6. Discussion

**Figure 3:**
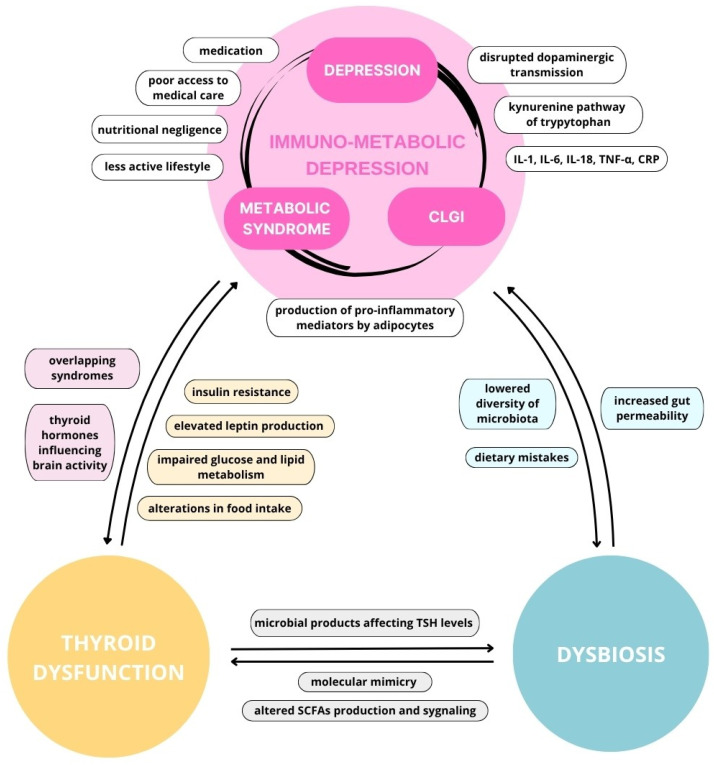
Overview of correlations between immuno-metabolic depression, thyroid dysfunction, and dysbiosis. IL-1—Interleukin 1, IL-6—Interleukin 6, IL-18—Interleukin 18, CRP—C-reactive protein, CLGI—Chronic low-grade inflammation, SCFAs—Short chain fatty acids, TD—Thyroid dysfunction, TNF-α—Tumor necrosis factor α, TSH—Thyroid-stimulating hormone.

Depression appears to arise from complex interactions between immune, metabolic, and neuroendocrine pathways, with growing evidence supporting a bidirectional link with metabolic disturbances. Gut microbiota and thyroid function emerge as important components of this network, potentially contributing to inflammation, metabolic dysregulation, and altered central nervous system function. Together, these interconnected mechanisms support a more integrative view of depression as a systemic, immuno-metabolic disorder involving the gut–thyroid–brain axis. The key relationships described in this review are summarized in Figure 3.

### 6.1. Clinical Implications

The integrative perspective of immuno-metabolic depression has several important clinical implications. First, it highlights the need for a more holistic approach to the diagnosis and management of depressive disorders, considering not only psychiatric symptoms but also metabolic, inflammatory, and endocrine status. This is particularly important given that patients with an immuno-metabolic profile of depression may respond less favorably to conventional antidepressant treatment alone, especially in the presence of elevated inflammatory markers, underscoring the need for more personalized and mechanism-based therapeutic strategies. Routine assessment of metabolic parameters, inflammatory markers, and thyroid function in patients with depression may improve identification of specific biological subtypes and enable more personalized treatment strategies.

Additionally, the bidirectional relationship between depression and metabolic disturbances suggests that interventions targeting lifestyle factors, such as diet and physical activity, may have dual benefits for both mental and metabolic health. Increasing evidence indicates that behavioral interventions, including structured exercise programs, can modulate inflammatory pathways and improve depressive symptoms, supporting their role as an adjunct to pharmacological treatment.

Finally growing interest in the gut microbiota–thyroid–brain axis opens new avenues for therapeutic interventions. Although still at an early stage, modulation of gut microbiota through dietary interventions, probiotics, prebiotics, or synbiotics may represent a promising complementary strategy in the management of depression.

### 6.2. Course of Future Studies

Future research should focus on clarifying the causal mechanisms linking metabolic, inflammatory, endocrine, and neurobiological pathways in immuno-metabolic depression. In particular, there is a need for well-designed longitudinal and interventional studies that integrate multiple levels of analysis, including clinical, biochemical, and microbiome-related data. Such an approach would enable the distinction between cause and effect, which remains one of the main limitations of current research. Moreover, standardization of methodologies, especially in microbiome research, is essential to improve reproducibility and comparability across studies. The analysis of microbiota function may provide more biologically relevant information than its taxonomic structure alone. Additionally, identifying reliable biomarkers of immuno-metabolic depression may facilitate earlier diagnosis and more targeted therapeutic approaches. Potential biomarkers include inflammatory markers, markers of intestinal permeability, microbiota-derived metabolites, and thyroid function parameters. 

Importantly, future approaches may benefit from the development of integrated biomarker indices that combine these domains, enabling a more comprehensive characterization of the immuno-metabolic profile. This strategy could allow an earlier identification of patients with immuno-metabolic depression and support the development of more personalized therapeutic strategies. In addition, future research should also deepen the analysis of the concept of brain hypothyroidism, particularly in patients with normal serum thyroid hormone levels. The application of advanced neuroimaging techniques, combined with analysis of peripheral and central biomarkers, may help determine whether a local deficiency of thyroid hormones in the brain contributes to the onset of depressive symptoms and treatment resistance. Finally, integrative, multi-omics approaches may provide deeper insight into the complex interactions underlying this condition and support the development of mechanism-based treatments. Further development of this field requires moving beyond isolated, cross-sectional analyses toward multidimensional, longitudinal, and mechanistic research models.

### 6.3. Study Limitations

This review has several limitations that should be acknowledged. First, because of its narrative design, the study may be subject to selection bias, as article inclusion was not based on a systematic protocol. Second, the included literature is heterogeneous, comprising systematic reviews and meta-analyses, longitudinal cohort studies, cross-sectional studies, and mechanistic and experimental research. This variability in study design, populations, and outcome measures limits direct comparability and precludes quantitative synthesis.

Furthermore, a substantial proportion of the included evidence is observational, including large cohort studies and epidemiological analyses, which limits the ability to infer causal relationships between depression, metabolic disturbances, thyroid dysfunction, and gut microbiota. While some mechanistic insights are derived from experimental and animal studies, their generalizability to human populations remains limited. Additionally, several included studies rely on indirect markers (e.g., inflammatory biomarkers, microbiota composition, or hormonal levels), which may not fully capture the complexity of the interactions under investigation. Finally, the search was restricted to selected databases and to studies published in English, which may have resulted in the omission of relevant research.

## 7. Conclusions

In light of the available evidence, depression can be viewed as a complex disorder with an immuno-metabolic background, in which interactions between the immune system, metabolism, gut microbiota, and thyroid function play a significant role. Accumulating evidence indicates a bidirectional relationship between depression and metabolic syndrome, with each condition potentially exacerbating the other through shared inflammatory and metabolic pathways. An important component of this system is the gut microbiota, which, while essential for maintaining homeostasis, may also contribute to chronic low-grade inflammation and metabolic dysregulation when altered. Within this multifactorial framework, this review highlights thyroid dysfunction as an additional, often underappreciated element linking these systems. Thyroid hormones interact closely with metabolic, immune, and central nervous system processes, suggesting that even subtle alterations in thyroid function may contribute to depressive symptomatology. Further longitudinal and interventional studies are needed to clarify the directionality and mechanisms of the aforementioned disturbances. Such efforts may support the development of more effective and personalized therapeutic strategies for immuno-metabolic depression.

## Figures and Tables

**Table 1:** Overview of characteristic changes in gut microbiota composition and related biomarkers across selected clinical conditions, based on current literature.

	Dysbiosis	Leaky Gut	Inflammation	Thyroid Disfunction
Depression	↓ Gut SCFAs [79] ↓ Gut neurotransmitters [94] ↓ Microbiota diversity	↑ Markers of intestinal permeability (LPS, I-FABP, LBP, zonulin) [67,95]	↑ Proinflammatory cytokines [96]	↑ Leptin [97] Altered neurotransmitters production Altered TSH secretion [82]
Abdominal obesity	Altered microbiota composition (↓ A. muciniphila, ↑ F/B ratio) [25,30]	↑LPS	↑ Proinflammatory cytokines [23]	↑ Leptin ↑ fT3 [98]
Thyroid disfunction	↓ *Lactobacillus*, *Bifidobacterium* [78] ↓ Treg cells [71]	↑ Zonulin [99] ↑ LPS [80]	Altered conversion of T4 into T3 [100]	n/a

↑—elevated, ↓—decreased. SCFAs—Short chain fatty acids, LPS—Lipopolysaccharide, I-FABP—Intestinal fatty acid binding protein, LBP—Lipopolysaccharide binding protein, TSH—Thyroid-stimulating hormone, fT3—free triiodothyronine, T4—thyroxin, T3—triiodothyronine, F/B ratio—Firmicutes/Bacteroidetes ratio, Treg cells—Regulatory T cells.

## Data Availability

No new data were created or analyzed in this study. Data sharing is not applicable to this article.

## Abbreviations

The following abbreviations are used in this manuscript:

AITD	Autoimmune thyroid disease
APC	Antigen-presenting cells
BMI	Body Mass Index
CLGI	Chronic low-grade inflammation
CRP	C-reactive protein
CSF	Cerebrospinal fluid
F/B	Firmicutes/Bacteroidetes ratio
fT3	Free triiodothyronineA
fT4	Free thyroxine
HPA axis	Hypothalamic-Pituitary-Adrenal axis
HPT axis	Hypothalamic-Pituitary-Thyroid axis
HT	Hashimoto’s thyroiditis
I-FABP	Intestinal fatty acid-binding protein
IL-1	Interleukin 1
IL-18	Interleukin 18
IL-6	Interleukin 6
IMD	immuno-metabolic depression
LPS	Lipopolysaccharide
MDD	Major Depressive Disorder
MetS	Metabolic Syndrome
MS	Metabolic Syndrome (or context-dependent)
NIS	Sodium/Iodide Symporter
ROS	Reactive oxygen species
SCFAs	Short-chain fatty acids
TH1	T-helper cell type 1
TNF-α	Tumor necrosis factor α
TRH	Thyrotropin-releasing hormone
TSH	Thyroid-stimulating hormone
VLDL	Very low-density lipoprotein
WC	Waist circumference
